# “Treated like a Human Being”: perspectives of people who inject drugs attending low-threshold HCV treatment at a syringe service program in New York City

**DOI:** 10.1186/s12954-023-00831-9

**Published:** 2023-07-27

**Authors:** Shashi N. Kapadia, Yesenia Aponte-Melendez, Alicia Rodriguez, Melinda Pai, Benjamin J. Eckhardt, Kristen M. Marks, Chunki Fong, Pedro Mateu-Gelabert

**Affiliations:** 1 Division of Infectious Diseases, Weill Cornell Medicine, 1300 York Avenue Ste A-421, New York, NY 10065 USA; 2 Department of Community Health and Social Sciences, CUNY Graduate School of Public Health and Health Policy, 55 W 125th Street, New York, NY 10027 USA; 3 New York University Rory Meyers College of Nursing, 433 First Avenue, New York, NY 10010 USA; 4 Division of Infectious Diseases, New York University Grossman School of Medicine, 550 First Avenue, New York, NY 10016 USA

**Keywords:** Hepatitis C, Clinical trial, Treatment uptake, Stigma, Low-threshold, Substance use disorder

## Abstract

**Background:**

Hepatitis C virus (HCV) treatment can effectively cure HCV among people who inject drugs (PWID). Perspectives of PWID treated in innovative models can reveal program features that address barriers to treatment, and guide implementation of similar models.

**Methods:**

We interviewed 29 participants in the intervention arm of a randomized trial. The trial enrolled PWID with HCV in New York City from 2017 to 2020 and tested the effectiveness of a low-threshold HCV treatment model at a syringe services program. Participants were purposively sampled and interviewed in English or Spanish. The interview guide focused on prior experiences with HCV testing and treatment, and experiences during the trial. Interviews were inductively coded and analyzed using thematic analysis.

**Results:**

Before enrollment, participants reported being tested for HCV in settings such as prison, drug treatment, and emergency rooms. Treatment was delayed because of not being seen as urgent by providers. Participants reported low self-efficacy, competing priorities, and systemic barriers to treatment such as insurance, waiting lists, and criminal-legal interactions. Stigma was a major factor. Treatment during the trial was facilitated through respect from staff, which overcame stigma. The flexible care model (allowing walk-ins and missed appointments) helped mitigate logistical barriers. The willingness of the staff to address social determinants of health was highly valued.

**Conclusion:**

Our findings highlight the need for low-threshold programs with nonjudgmental behavior from program staff, and flexibility to adapt to participants’ needs. Social determinants of health remain a significant barrier, but programs’ efforts to address these factors can engender trust and facilitate treatment.

*Trial registration* NCT03214679.

## Introduction

Hepatitis C virus infection (HCV) is a common chronic infection that, in the United States, primarily affects people who inject drugs. HCV leads to death from liver complications, although the time frame for clinical morbidity from HCV measures in years [22]. There has been a recent transformation in HCV care and outcomes, driven by the invention of direct acting antiviral (DAAs), which are highly effective, well-tolerated, oral treatment regimens. DAAs have greatly improved upon the previous standard of care, which was injection interferon. Compared to interferon, DAAs are better tolerated, more effective, and simpler to administer [1]. The use of DAAs has led to optimism that widespread treatment can enable HCV elimination, especially if treatment is targeted towards people who inject drugs (PWID) [15].

Despite the hope of HCV elimination, treatment uptake among PWID has been limited. Data from Baltimore in 2018, for instance, a full 4 years after DAAs were approved, showed only 26% treatment uptake among PWID, suggesting a significant missed opportunity [9]. This is likely to be driven by several factors. First, the asymptomatic nature of HCV means that identification of the disease relies on successful screening, which may not successfully reach the intended populations. Additionally, the medical complications of the disease are sufficiently distant that treatment may not be a priority [3, 28]. Second, PWID who received a diagnosis before the new DAA medications may not have been aware of these new medications, thus delaying treatment uptake. Third, insurance policies often hamper treatment access by necessitating sobriety or drug screening before providing coverage for treatment [19]. Finally, PWID experience significant stigma, both internal stigma related to their disease, and enacted stigma within the healthcare system. Healthcare-related stigma has compounding effects: it makes PWID less likely to disclose injection behaviors, less likely to seek healthcare, and engenders mistrust with healthcare providers [5, 25]. Simultaneously, healthcare providers can perpetuate stigma, potentially making them less likely to offer treatment to PWID, or to impose structures that make treatment for PWID more difficult, such as requiring multiple appointments or proof of sobriety [5, 12].

A recent study that asked PWID about their preferences for HCV treatment found that comprehensive patient-centered care that is attentive to their needs and competing priorities would be critical for enabling successful treatment completion [3]. A set of care strategies termed “low-threshold” healthcare aligns with these priorities, though it has primarily been described in reference to substance use disorder treatment [17]. There are several international examples of low-threshold HCV care that are shown to be effective, though not all are labelled as such [10]. For example, an HCV clinic based in a harm reduction program in Oslo achieved HCV cure in 90% of 340 PWID [24]. Similar successful models are reported from Australia and the United Kingdom [27, 33]. Notably, each of these models involved close partnership with syringe service programs (SSPs) as treatment sites. Beyond their primary function of providing injection equipment, SSPs are key partners in multiple health, educational, and social interventions to improve the lives of PWID. In 2020, US survey of 152 SSPs, almost 60% of programs offered HCV testing and 14% HCV treatment [4]. Despite this, descriptions of low-threshold SSP-based HCV-treatment models are rare in the US, and limited to clinical studies that did not report on patient perspectives [2, 8].

In the current study, we report data collected from PWID enrolled in a randomized clinical trial of a low-threshold HCV treatment model that was co-located at a syringe service program (SSP). Called the Accessible Care model, the intervention was designed to identify and eliminate barriers to treatment among PWID. The Accessible Care model was designed informed by qualitative interviews with PWID, clinicians, and other service providers. The model was piloted at a single SSP in New York City, and then implemented at a second site after adaptation; and in both cases found to be effective at providing HCV cure for SSP clients [7, 8]. The healthcare model included features that have since been described as low-threshold healthcare: including flexible appointments, allowing walk-ins, accepting a wide range of public insurance plans, a non-judgmental and non-punitive philosophy, and willingness to address structural and social barriers to access. We conducted qualitative interviews with trial participants after they had initiated HCV treatment in the trial. We sought to understand their perceived barriers to treatment prior to the study, and how enrollment in the Accessible Care model facilitated successful treatment completion.

## Methods

### Objective

The current analysis aims to report on qualitative experiences with HCV testing and treatment among a sample of PWID in New York City with HCV.

### Study population and setting

We conducted semi-structured qualitative interviews of participants enrolled in a randomized controlled trial of HCV treatment co-located at a syringe service program (SSP) in New York City. The trial enrolled adult participants who were HCV RNA positive, and had injected drugs for at least one year, and at least once in the past 90 days; excluding those with decompensated cirrhosis, pregnant women, and those who were engaged in HCV care in the past 6 months. Participants were randomized 1:1 to receive an intervention (called “Accessible Care”) or “Usual Care.” Accessible Care participants were offered a medical visit with a physician located at the SSP, and after medical evaluation, were offered HCV treatment at the SSP. A study care coordinator facilitated participants’ treatment by providing adherence support and logistical support in keeping medical appointments, and also delivered a reinfection prevention training session during treatment. Participants randomized to Usual Care were referred to local providers, via a HCV patient navigator employed by the SSP. The primary outcome was sustained virological response at 12 weeks after treatment (SVR12), which is equivalent to a cure of HCV. The trial results are published elsewhere [7]. Briefly, of 165 enrolled participants, 55/82 (67%) of the Accessible Care arm had HCV cure by 12 months follow-up, compared to 18/83 (23%) of the usual care arm. The difference in response rates was largely due to differences in treatment initiation in the two arms (90% and 71% respectively), for those who initiated treatment HCV cure was similar in both the Accessible Care and usual care arms (86% in both arms).

### Qualitative subsample

This manuscript reports results from qualitative interviews with 29 participants, all of whom were randomized to the Accessible Care intervention. All participants who were interviewed had initiated treatment during the trial, but not all had completed treatment. Participant characteristics of the qualitative sample are shown in Table 1. Participants were selected with the goal of achieving diversity in age, gender, ethnicity, and housing status. While no participant declined interview, we were unable to interview participants who did not initiate treatment as the majority of those were lost to clinical follow up or not accessible to the study team (e.g. incarcerated).

**Table 1:** Participant characteristics of qualitative sample

	Qualitative study participants (*n* = 29)
*Demographics*	
Mean age in years (SD)	43.1 (9.8)
Gender	
Male	23 (79%)
Female	6 (21%)
Transgender	0 (0%)
Ethnicity	
Hispanic	18 (62%)
Not Hispanic	11 (38%)
Race	
Black	4 (14%)
White	6 (21%)
Multi-racial	19 (65%)
Other groups or refused	0
Birthplace	
United States	19 (65%)
Puerto Rico	8 (28%)
Other	2 (7%)
*Socioeconomic factors*	
Homeless in past 3 months	16 (55%)
Ever incarcerated	26 (90%)
Employment	
Unemployed	25 (86%)
Disability	3 (10%)
“Off-books” employment	1 (3%)
*Healthcare engagement before study*	
Aware of HCV diagnosis	25 (86%)
Referred for HCV treatment	17 (59%)
Ever taken HCV treatment	6 (21%)
Seen a doctor in the past 3 months	21 (72%)
Drug treatment in past 3 months (overall)*	26 (90%)
Methadone	20 (69%)
Buprenorphine	1 (3%)
Residential treatment	10 (34%)
*Study outcomes*	
Initiated treatment	29 (100%)
Completed treatment	27 (93%)
Achieved SVR12**	27 (93%)

*SVR12* sustained virologic response at 12 weeks

*Total does not sum to 29 because participants could receive multiple forms of drug treatment in past 90 days. **3 participants did not achieve SVR12 during initial treatment but did so after re-treatment while still engaged in the study

### Data collection

We conducted semi-structured in-person interviews with each participant. Interviews were conducted by trained staff and digitally audio-recorded; the interviews were conducted in a private room at the SSP and ranged in length from 60 to 90 min. Interviews focused on participants economic and housing status, drug use history, previous experiences with HCV testing and treatment, stigma, and experiences of HCV care at the SSP. Participants’ demographic information and HCV history was collected in quantitative questionnaires administered upon enrollment (Table 1). Interviews were conducted in English (*n* = 20) and Spanish (*n* = 9) according to participants’ preferred language. All study procedures were approved by the Institutional Review Board of Weill Cornell Medical College. Each participant provided written consent and was compensated $50.

### Analysis

Interviews were recorded and transcribed. English-language interviews were transcribed verbatim by a consultant; the Spanish-speaking interviews were transcribed by the study’s native Spanish-speaking Research Assistant. Interviews were coded independently by 3 researchers (SK, YA, AR) using a content analysis approach, in which data excerpts were summarized into codes, which were subsequently categorized into themes [16]. Additionally, researchers created memos during the analysis to record insights. The codebook was determined inductively, although researchers were informed by our previous quantitative work on HCV treatment and stigma [11, 18, 20]. Interviews were analyzed in English or Spanish by native bilingual research staff. All quotes are reported in English. Analyses were conducted using DeDoose version 9.0.46 [29].

## Results

We report results that pertain to 2 broad domains: (1) Participants’ previous experiences and barriers faced accessing HCV treatment; and (2) How the Accessible Care clinical model addressed previous barriers.

### Domain 1: Participants previous experiences and barriers faced accessing HCV treatment

None the participants enrolled in this study were engaged in HCV treatment at the time of enrollment, but many were aware of their diagnosis and some had attempted treatment (Table 1). The data about their HCV experience before the study reveal a trajectory from diagnosis to treatment that is often drawn out because of fragmented healthcare, structural barriers to treatment, stigma, and a lower prioritization of HCV care for both patients and providers.

#### Theme 1: Lack of urgency for HCV treatment from providers

Participants who were aware of their diagnosis prior to enrollment (*n* = 27) often reported being diagnosed during interactions outside of the traditional settings in which hepatitis C is treated. While most participants reported having a primary care provider (*n* = 18) and many attended methadone maintenance programs (*n* = 20), these providers were rarely the source of HCV diagnosis. Instead, most participants were diagnosed in alternative settings, such as incarceration (*n* = 6), inpatient drug treatment (*n* = 5), prenatal care (*n* = 2), or while attending syringe service programs (*n* = 5).

I tested positive…when I went up north, when I went in to prison. Before they put you into population they make sure you do not have any communicable diseases and that is how I found out I had HCV. [That was] 20 years ago. (60-year-old male)

Follow-up care for HCV was inconsistent and often delayed. The participant quoted above did not receive HCV care until 20 years after his diagnosis, despite multiple healthcare interactions in between. Another participant diagnosed during incarceration was given a list of providers after release, 2 years after his diagnosis, but did not seek treatment until enrolling in the clinical trial (43-year-old male).

Participants who did access medical care reported decreased urgency of HCV treatment due to ambivalence on the part of doctors. Most participants who experienced this cited that their blood testing did not indicate urgent HCV treatment at that time: “doctors said…my blood work it wasn’t right…it’s not chronic.” (41-year-old female). One participant was told in jail that he did not need treatment yet because of a normal liver test, which was the standard practice in 1995 when he was diagnosed. However, in 2018, at a treatment program in New York, he was referred to treatment but did not attend the appointment because “I didn’t know about [HCV], what it can cause, what can happen to you…[They told me] that there is a treatment, but it was not mandatory for me because my liver was not that bad” (46-year-old-male).

#### Theme 2: Competing priorities for patients

Participants who were referred to treatment providers (*n* = 17) often faced competing priorities that affected their ability to engage with those providers. For one, the threat of lost income from working prevented the participant from attending medical visits: “the job I was working back then…for me to take a day off would have been…in my mind, the money was more important” (46-year old male). Another participant had caretaking responsibilities with family around the time of HCV diagnosis: “I didn’t pursue a doctor. I was going through a lot of things at the time. My daughter was very sick, my wife was sick, my mother was sick. I was in Florida, trying to maintain life here and in Florida” (57-year-old-male). Additionally, though participants did not explicitly mention drug use as a competing priority, several had the impression or had heard from providers that “if you use you can’t get [the treatment],” leaving them with the understanding that they would have to choose between continuing drug use and engaging in HCV treatment.

#### Theme 3: Self-efficacy in accessing healthcare

Few participants explicitly mentioned substance use disorder, mental health, or homelessness as a reason for not seeking treatment. One participant, who was referred to HCV treatment since in the 1990s, was “too involved in the street life, so I didn’t have an interest in it” (52-year-old male). Even when not explicitly connecting to their drug use, several participants expressed low self-efficacy, meaning they doubted their own capacity to engage in HCV treatment despite understanding the benefits. For example, one participant recounted:

It’s insane how I didn’t get treatment. I mean at first, I wasn’t taking no interferon…but they had this other medication for years and years and my family kept telling me, people kept telling me: ‘yo why don’t you get that fixed, what’s wrong with you man?’ ‘I’m in the streets, y’know. I’ll do it, I’ll do it, I’ll do it.’ It’s always tomorrow. (45-year-old male).

Another participant had deferred treatment for many years in the interferon era, and was interested in starting treatment when they learned about new medications, but was deterred by the barrier of travelling to medical visits:

There was one time…they had referred me to some place on Madison Avenue. But I never went… I’m bad at appointments…Other boroughs, go here, go there, I will not. I mean, it’s in my own best interest, but it won’t happen” (50-year-old male).

One participant reported not seeking medical care after learning about their HCV because “I didn’t care. I had nothing to live for at the time anyway, so what the f***” (45-year-old female).

#### Theme 4: Systemic barriers to healthcare access

Only 17 of the 29 participants had been referred to treatment before the study, and fewer still (*n* = 6) participants had tried to follow-up on a referral to a treatment provider (Table 1). Those that did reported systemic barriers to treatment. These included: (1) limitations in providers accepting health insurance (2) limitations in providers accepting new patients, (3) the need for multiple appointments, (4) needing to travel a long distance to find a provider, and (5) criminal-legal interactions interrupting healthcare access. For example, one participant was referred by their primary care provider to a specialist, but that provider’s office needed multiple insurance verifications to book an appointment and the participant was ultimately not able to see that provider (40-year-old male). Another was already an established patient at a treatment center but could not receive HCV treatment because of a long waiting list (52-year-old male). One participant was dissuaded from seeking treatment from the anticipation of transportation difficulties and paperwork: “[I didn’t go to the appointment] because I lived in the Bronx and this was over in Manhattan, and then they would ask for a mountain of things… I imagine that you would have to fill out papers, have to take my blood” (46-year-old male). One participant had successfully seen a doctor but needed “like 4 doctors’ visits before anything even happened, and then you gotta get tested for this and that before they can even call the insurance company and try to get approved. And then it takes months to even get approved” (28-year-old male). This participant did successfully receive a prescription, but “wound up getting arrested…and never started taking the medication” (28-year old male).

#### Theme 5: Stigma

When asked how doctors treat people who use drugs in general, a common perception among participants was that doctors perceived PWID as less worthy. Participants described numerous ways in which their perception of unworthiness as patients manifested: ranging from frank disrespect to decreased attentiveness to medical care: “Sometimes, they attend others faster and better than those who use drugs. For me it’s always indifference. I am not saying that every doctor is the same, but the majority “*te tratan con el codo”* [treat you poorly] when they know you are addicted to drugs. That’s the reality” (39-year-old male). Several participants perceived a change in their doctors’ feelings after revealing drug use behavior to them: “I feel judged… I noticed when I told them I had HCV and was cured from it and that I was in a [methadone] program…their whole way of talking to me and treating me changed” (47-year-old male). Another shared an experience of a distinct change in demeanor: “One time, before I said I was using drugs, [the medical provider] would come in, with no gloves, check my throat, just normal things, and when I said I was using, right away he put his gloves on and made me feel uncomfortable. Just the demeanor and body language, just everything changed” (41-year-old female).

Another recounted: “90% of [doctors and nurses] believe that you are trash, crap, and they don’t even want to touch you…many times you share symptoms you are experiencing and they don’t even check you. As soon as you tell them that you use drugs, they don’t even check you. They even give you prescriptions…to let you go quickly” (54-year-old male) A similar experience was described by another participant as “They treat us like we’re half-humans…they don’t look at how the symptoms might be related to other diseases. They just like, well, you’re a junkie and that’s it” (45-year-old female). Another felt that their doctor even questioned the intentions of their medical related questions, “thinking the things I’m asking him for is because of some underhanded reason, not for my personal care” (40-year-old male).

For some participants, the experience of stigma was explicitly linked to why they did not seek or receive HCV treatment: “They [doctors] didn’t want to touch me! Hepatitis was just as evil as HIV, if not more” (45-year-old female). For others, the perception of stigma discouraged healthcare interaction except in emergency settings, which by extension would limit the ability to receive HCV treatment, which is provided entirely in outpatient settings.

Internalized stigma was also a major component of participants’ feelings about HCV and drug use. One participant felt that doctors treat people who use drugs differently “because [they] also behave differently… when I am using drugs… I act like not a human sometimes, but I am still a human being” (28-year old female).

The common experience of stigma was sometimes tempered by positive interactions with healthcare providers: “I’ve been lucky in that I found my primary care physician is…really open-minded, very understanding” (35-year-old female). One participant described his/her perception of doctors who care as follows: “I think it’s a 60/40 split. I think 60% genuinely care and 40% look down and don’t give them the best treatment” (46-year-old male).

### Domain 2: How the accessible care clinical model addressed previous barriers

**Fig. 1:**
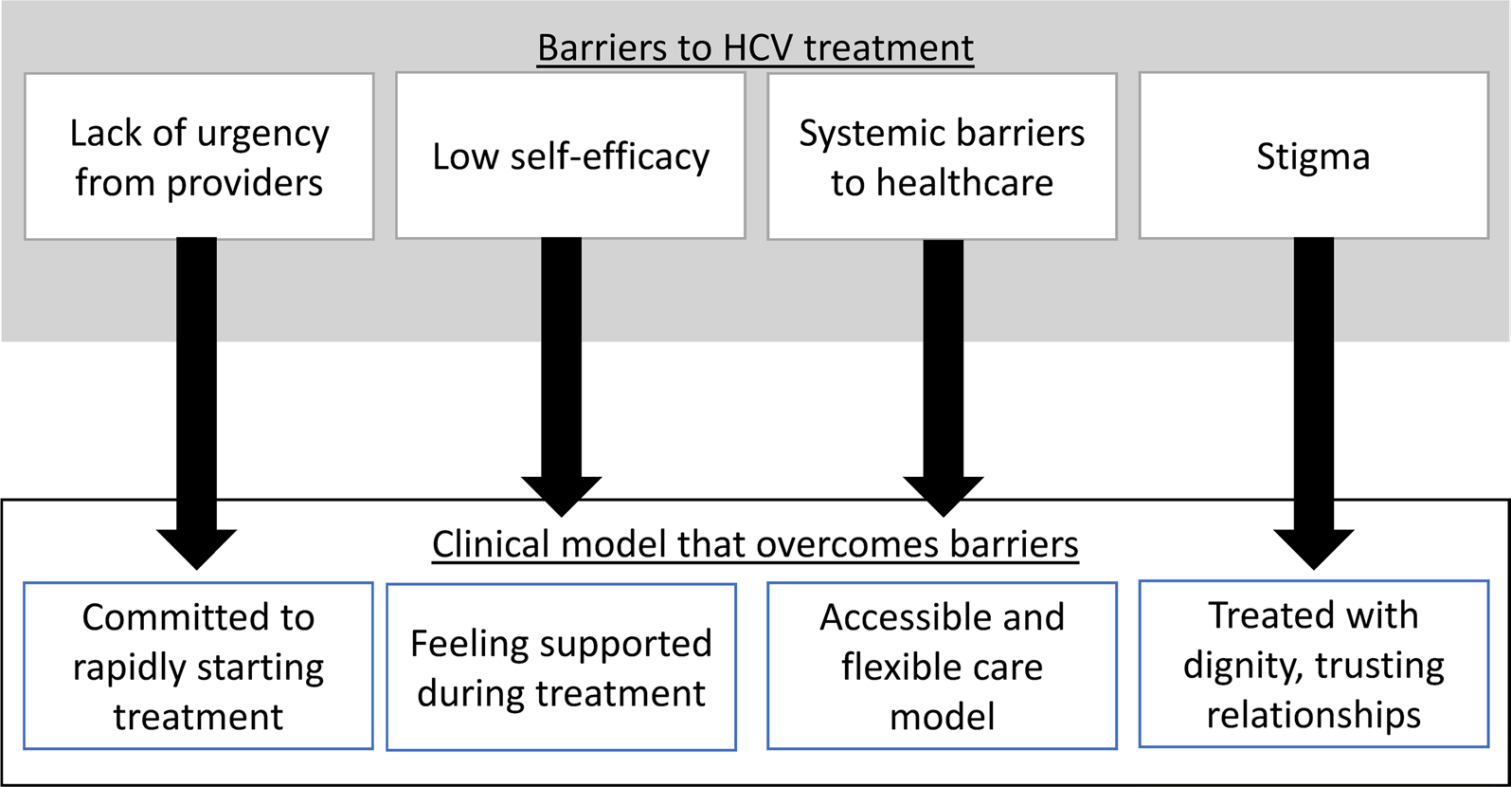
Barriers to HCV treatment reported by people who inject drugs and clinical program features that helped overcome them

Figure 1 shows the relationship between barriers participants experienced before enrollment and perceptions on how those barriers were overcome during treatment in the clinical trial.

#### Theme 6: Commitment to HCV treatment and cure

In contrast to the transient interactions and lack of urgency in their previous experiences, participants reported an urgent commitment to providing HCV treatment and achieving a cure. Though this would be expected in the context of a clinical trial, the participants’ favorable responses to this urgency contrasted with experiences at other providers: “I was diagnosed with Hep C and the doctor told me we could…get you medicated and on the treatment as quick as possible…It was a pretty quick process. Most of the time…knowing friends who had it, they had to wait quite some time, some a year, some less” (47-year-old male).

The providers’ commitment to initiating treatment, rather than an ambivalent approach, led to increased commitment from participants to start treatment. One participant overcame an initial doubt that their disease was even curable:

In reality, when I started I didn’t have faith, I thought the medication was bullshit…When I came here and they offered me treatment, I thought that my liver was done, you know. I took it because I had nothing to lose. I did not know 100% I would be cured…When [the doctor] told me “your liver is fine. If you take your pills 100%, your hep C is going to go away.” I liked his optimism when he was speaking to me. I completely put my faith in it and started to take the medicine prescribed. And thank God it came out 100% positive (39-year-old male).

Another described the sentiment more succinctly: “He gave me a little oomph to get up and do something!. A little kick in the ass to get going, that’s what I needed!” (57-year-old male).

#### Theme 7: Feeling supported during the treatment period

During treatment, participants mentioned a feeling of support from study staff. This support encouraged adherence and overcame participants’ concerns about their ability to successfully complete the treatment course. Importantly, participants who were currently using drugs mentioned the misconception that they would not be able to take the HCV treatment while still using drugs, and the doctor’s correcting of that misconception: “he told me that if I took the pills the way I had to, we could let me use drugs as I want, that it wouldn’t affect my treatment” (39-year-old male).

One participant appreciated the study physician “calling and making sure I was taking the medicine and feeling okay” (54-year-old female). Another participant was grateful for the mode of delivery: “he set up the medication to arrive directly at my house, so I didn’t have to go and find it or anything like that” (39-year-old male). Encouraging participants to persist through side effects was another way in which the study physician encouraged treatment completion:

“For example, when I didn’t have good side effects from it, [the doctor] suggested to take it at a different time of day…I don’t think I could have handled feeling that tired and sick, and he said ‘Take it at nighttime, it will get better.’ If not for that I think I would have said ‘I’ll do it at a different time of my life.’ (25-year-old female)

Participants reported appreciation for the accessibility of the healthcare team: “If I needed to call to talk to them…they’ll be willing to let me in and say ‘Have a seat’” (47-year-old male). Another highlighted the ease of communication with the study doctor specifically, “he gave me his cell phone, said ‘call me any time you want, any concern’” (44-year-old male).

#### Theme 8: A flexible co-located care model that minimizes and addresses systemic barriers

The healthcare team was willing to work around participants’ competing priorities and work through logistical and systemic issues that would otherwise have been barriers to treatment completion. This was true even if the patient may have missed follow-up visits: “No matter how irresponsible I was with my appointments, being on time, they were always so patient with me and worked with me and re-scheduled me without making me feel like I messed up. That was so important” (46-year-old male). In this case, the participant placed their own behavior in a negative valence, but felt it was important that they were not made to feel that way by the provider.

In another example, the team obtained prior authorization for treatment, sometimes after a struggle, and this was seen as being a sign that the healthcare team was advocating on behalf of the participant: “[The doctor] will always go above and beyond. For instance, my medical plan was giving us a hard time to pay for the treatment, so he went and appealed it and just kept fighting and fighting until finally I got approved” (43-year-old male). For one participant when their medication pick-up from the pharmacy was interrupted, “[the study team] went all the way to my house to give me my medication” (32-year-old male). Even when participants faced social barriers that could not be solved, some expressed gratitude for the degree of effort: “I was homeless, okay… this is what’s so beautiful about [this doctor] and the people you work with, that I truly believe it ain’t only about the medication…[they] were like, ‘we gotta get him somewhere. He needs a place where he can rest, wash up, sleep, and take his medicine’” (47-year-old male).

#### Theme 9: Being treated with dignity enables forming trusting relationships and combats stigma

Participants highly valued how clinical staff treated them, and the language they used contrasted directly with the stigma and dehumanization experienced in past healthcare experiences: “I felt respected as a man, as a person, as a human being” (45-year-old male). Another stated “Like a person with respect. The most important thing is to treat a person with respect” (52-year-old male). The study physician was perceived as being patient, “not rushing me” (57-year-old male) and “very kind” (50-year-old male). The physician “doesn’t look at you no different if you’re a drug user” (48-year-old male). The care coordinator “made me feel totally comfortable” (46-year-old male) and “always showed her support that she’ll be there for me regardless (43-year-old male). The formation of a trusting relationship was an important facilitator to treatment completion and validated the participant’s choice to seek out HCV treatment: “Made me feel at ease, like I am doing the right thing for myself” (45-year-old male).

## Discussion

In this qualitative study of participants in the intervention arm of a randomized clinical trial of HCV treatment, barriers to HCV treatment among people who inject drugs were overcome by features of the “Accessible Care” treatment program. Despite the availability of easy-to-tolerate HCV treatment, even the participants who were aware of their HCV diagnosis faced significant barriers that in accessing a treatment provider that was receptive to the unique needs of this population. These same participants described features of the clinical trial program that supported them in overcoming these barriers and receiving HCV treatment. The trial itself was highly effective, as described elsewhere, with 67% of intervention arm participants achieving HCV cure compared to only 23% of participants in the “Usual Care” arm [7]. These qualitative findings underscore the features of the treatment intervention that participants identified as strongly supportive, which is critical for informing future implementation of similar interventions.

This trial recruited a population who was not engaged in HCV care at the time of enrollment, although many had previously sought medical care and even attempted curative treatment in the past. As the United States and other jurisdictions strive for HCV elimination, understanding the experiences of populations who have not yet received treatment is critical. Our findings show that from the perspectives of people infected with HCV, treatment is often not a priority for their healthcare providers. In these cases, both providers’ and participants’ own deferral of HCV treatment led to often long delays, despite participants being aware of the infection, and potentially a lack of urgency to do so. This finding corresponds with other studies in similar populations. For instance, Tofighi and colleagues write about a “benign perception of HCV” among patients admitted to detoxification centers, and highlight the impression that patients receive from providers that HCV treatment could always be addressed at some later time [31]. Madden et al. [23], interviewing PWID in Melbourne Australia, described this phenomenon as “waiting for the perfect moment” for treatment, and often motivated by messaging from providers to wait for disease progression, sobriety or new treatment approvals. Delayed treatment, while not always harmful for an individual patient, increases the opportunity for transmission events to occur, which is counterproductive to the goal of HCV elimination. In contrast to these experiences, our study found that participants responded to a sense of commitment from a trusted provider, which motivated the initiation and completion of HCV treatment without excessive delays. This experience serves to dispel the common myth that people who inject drugs are not interested in their own health, but instead respond to positive commitments from healthcare providers. A further understanding of the provider perspective of HCV treatment eligibility, and development of educational or intervention strategies aimed at reducing the delay between a patient’s diagnosis and the offer of treatment would be important for reducing the ongoing transmission of HCV.

The practical aspects of the program design highlighted by our participants, such as providing access to physician’s cell phones and allowing for walk-in or easily rescheduled appointments, reflect a “low-threshold” hepatitis C treatment model. The term “low-threshold” has more commonly been used to describe models of buprenorphine treatment delivery. Jakubowski and Fox described common features of “low-threshold” buprenorphine treatment as including (1) same-day or rapid treatment entry, (2) a harm reduction approach that did not focus on abstinence from drug use, (3) flexibility in scheduling and treatment delivery, and (4) its availability in non-healthcare settings [17]. Although the term “low-threshold” is less commonly applied to HCV treatment models in published literature, it is notable that many of the factors listed above have individually been found to be features of successful models. In addition to SSP-based models described above, clinical trial data has validated the use of flexible treatment delivery: minimal monitoring strategies have been successful, as well as more intensive ones such as patient navigation and directly observed therapy [6, 21, 30]. Clinical trial data have also repeatedly shown that people who are currently using drugs can achieve high treatment rates without the need to insist on abstinence [13, 14]. Our findings emphasize how the “low-threshold” concepts can contribute to the success of treatment programs by overcoming barriers in traditional healthcare systems.

In addition to the practical aspects of program delivery, participants emphasized how care providers and staff engendered trust and confidence by offering a non-judgmental approach to drug use, by listening and patiently answering questions, and by frequently checking-in to support the treatment process. These features of the healthcare staff stood in stark contrast to participants’ experiences at prior providers, where they would feel looked down upon or dismissed because of their drug use. A large body of literature has delineated the many ways that stigma manifests and hinders the health and social well-being of people who use drugs [32]. Stigma in the healthcare setting disincentivizes disclosure of drug use and results in delaying necessary healthcare [5]. In a quantitative analysis of participants from the study reported here, our team found that higher levels of enacted stigma were associated with decreased likelihood of seeking HCV treatment [11]. Our findings emphasize that practical aspects of treatment program design, while important, are insufficient to ensure successful engagement. It is equally necessary, if not moreso, for HCV treatment programs seeking to engage people who inject drugs to ensure that staff and providers treat patients with humanity and dignity, especially for a population whose prior experiences have led to an expectation of discrimination and disrespect. Achieving this aim is, in some sense, more difficult than setting a program’s entry criteria or appointment schedule. Past work has largely focused on achieving reduced stigma through some combination of educational interventions with healthcare staff: predominantly including presentation of information about a stigmatized condition and encouraging contact with members of the stigmatized group to foster connection [26]. However, there is little in the way of guidance for implementation and sustainability of these interventions, nor for consistent measurement of their effect, suggesting that a lot of work remains to provide compassionate care across the health system for PWID.

This study is one of the first to report qualitative data from participants in a co-located, low-threshold HCV treatment program, and the first we are aware of in the United States. Linking our data to participation in an effective clinical trial helps to emphasize the components of this intervention that most resonated with participants and were most identified as success factors.

Limitations include the single study site and urban location: similar interventions implemented in other settings, particularly rural areas, may have to contend with factors that were less emphasized here, such as transportation barriers. Also, as is the norm in qualitative research, the results here represent a thematic description of participants’ perspectives but should not be interpreted as an “average” response. Participants were recruited from a study that provided them with treatment, and as such their perspectives may differ from those of participants who have not engaged in treatment at all. The interviewers were not part of the clinical intervention but were associated with the study team, and this may have discouraged participants from being critical of the program. Furthermore, the participants in the study who did not initiate treatment were often lost to follow up or otherwise inaccessible for interview, and thus their perspectives are not represented here. Perspectives of PWID for whom this kind of treatment model is not successful would be beneficial in determining additional barriers that would need to be solved to achieve a universal treatment rate.

In summary, we found that people who inject drugs with HCV face substantial barriers to treatment, including ambivalence from providers, low self-efficacy, competing priorities, logistical barriers, and stigma. A flexible, accessible HCV treatment model that is committed to supporting participants through treatment initiation and completion, and to treating them without judgment and with respect, can successfully overcome these barriers to support HCV cure for this marginalized population.

## Data Availability

Qualitative data from this study will not be made publicly available, due to the sensitive nature of the topics discussed and the difficulty of fully de-identifying qualitative data from its context. Relevant excerpts can be made available upon reasonable request to the corresponding author in a manner consistent with the informed consent signed by participants.
